# Supplementary material for: Associations between regional blood-brain barrier permeability, aging, and Alzheimer’s disease biomarkers in cognitively normal older adults
Source: PLoS One. 2024 Jun 5;19(6):e0299764. doi: 10.1371/journal.pone.0299764 (PMC11152304; doi:10.1371/journal.pone.0299764)
Supplement: S1 Table — Table listing the 37 FS Desikan-Killiany atlas ROIs used for analysis and their abbreviations. (DOCX) [file pone.0299764.s001.docx]

|  | **ROI** | **Abbreviation** |  | **ROI** | **Abbreviation** |
| --- | --- | --- | --- | --- | --- |
| **Frontal** |  |  | **Temporal** |  |  |
|  | Caudal middle frontal | CMF |  | Amygdala | Amyg |
|  | Frontal pole | FPol |  | Banks of the superior temporal sulcus | BanksSTS |
|  | Lateral orbitofrontal | LOrF |  | Entorhinal | EC |
|  | Medial orbitofrontal | MOrF |  | Fusiform | Fu |
|  | Pars opercularis | Op |  | Hippocampus | HC |
|  | Pars orbitalis | Or |  | Inferior temporal | IT |
|  | Pars triangularis | Tr |  | Middle temporal | MT |
|  | Rostral middle frontal | RoMF |  | Parahippocampus | PHC |
|  | Superior frontal | SF |  | Superior temporal | ST |
|  | Paracentral | PaC |  | Temporal pole | Tpol |
|  | Precentral | PreC |  | Transverse temporal | TrT |
|  | Caudal anterior cingulate | CAC | **Parietal** |  |  |
|  | Rostral anterior cingulate | RoAC |  | Inferior parietal | IP |
| **Occipital** |  |  |  | Isthmus cingulate | IstCg |
|  | Cuneus | Cu |  | Postcentral | PoC |
|  | Lateral occipital | LO |  | Posterior cingulate | PCC |
|  | Lingual | Lg |  | Precuneus | PreCu |
|  | Pericalcarine | PerCa |  | Superior parietal | SP |
| **Other** |  |  |  | Supramarginal | SM |
|  | White matter | WM |  |  |  |
|  | Insula | Ins |  |  |  |
