# Supplementary material for: Associations between regional blood-brain barrier permeability, aging, and Alzheimer’s disease biomarkers in cognitively normal older adults
Source: PLoS One. 2024 Jun 5;19(6):e0299764. doi: 10.1371/journal.pone.0299764 (PMC11152304; doi:10.1371/journal.pone.0299764)
Supplement: S2 Table — ROI = region of interest. See S1 Table for a ROI abbreviation key. (DOCX) [file pone.0299764.s002.docx]

| **ROI** | **Dimension 1** | | **Dimension 2** | | **Dimension 3** | |
| --- | --- | --- | --- | --- | --- | --- |
|  | **K_trans_** | **PiB** | **K_trans_** | **PiB** | **K_trans_** | **PiB** |
| **Temporal** |  |  |  |  |  |  |
| Amyg | 0.60 | 0 | -0.44 | -0.38 | 0 | 0 |
| BanksSTS | 0 | 0.08 | 0 | 0 | -0.49 | -0.03 |
| EC | 0 | 0 | 0 | 0 | 0 | 0 |
| Fu | 0 | 0 | 0 | 0 | -0.14 | 0 |
| HC | 0 | 0 | 0 | -0.53 | -0.59 | 0 |
| IT | 0 | 0.20 | 0 | 0 | -0.22 | -0.13 |
| MT | 0.39 | 0 | 0 | 0 | 0 | 0 |
| PHC | 0 | 0 | 0 | 0 | 0 | 0 |
| TrT | 0.50 | 0 | -0.64 | -0.51 | 0 | 0 |
| **Occipital** |  |  |  |  |  |  |
| Cu | 0 | 0 | 0.24 | -0.01 | 0 | 0 |
| LO | 0 | 0 | 0 | 0 | 0 | 0 |
| Lg | 0 | 0 | 0 | -0.01 | 0 | 0 |
| PerCa | 0 | 0 | 0 | -0.14 | -0.27 | 0 |
| **Parietal** |  |  |  |  |  |  |
| IP | 0 | 0.41 | 0 | 0 | -0.52 | -0.37 |
| IstCg | 0 | 0 | 0.09 | -0.13 | 0 | 0 |
| PCC | 0.08 | 0.11 | 0.13 | 0 | 0 | -0.06 |
| PreCu | 0 | 0.37 | 0.17 | 0 | 0 | -0.21 |
| **Frontal** |  |  |  |  |  |  |
| PaC | 0 | 0.72 | 0.53 | 0.53 | 0 | -0.54 |
| Op | 0.26 | 0.35 | 0 | 0 | 0 | -0.68 |
| **Other** |  |  |  |  |  |  |
| Ins | 0.40 | 0 | 0 | -0.01 | 0 | -0.22 |
