# Supplementary material for: Associations between regional blood-brain barrier permeability, aging, and Alzheimer’s disease biomarkers in cognitively normal older adults
Source: PLoS One. 2024 Jun 5;19(6):e0299764. doi: 10.1371/journal.pone.0299764 (PMC11152304; doi:10.1371/journal.pone.0299764)
Supplement: S3 Table — PVC = partial volume corrected; ROI = region of interest. See S1 Table for a ROI abbreviation key. (DOCX) [file pone.0299764.s003.docx]

| **ROI** | **Dimension 1** | | **Dimension 2** | | **Dimension 3** | |
| --- | --- | --- | --- | --- | --- | --- |
|  | **K_trans_** | **FTP (PVC)** | **K_trans_** | **FTP (PVC)** | **K_trans_** | **FTP (PVC)** |
| **Temporal** |  |  |  |  |  |  |
| Amyg | 0 | -0.02 | 0.38 | 0 | 0 | 0 |
| BanksSTS | 0 | 0 | 0 | 0.69 | 0.25 | 0.07 |
| EC | 0.57 | -0.01 | 0 | 0 | 0 | 0 |
| Fu | 0.33 | 0 | 0 | 0.31 | 0 | 0.23 |
| HC | 0 | 0 | 0.42 | 0 | 0 | 0 |
| IT | 0 | 0 | 0 | 0.60 | 0.21 | 0.33 |
| MT | 0 | 0 | 0.18 | 0.05 | 0.1 | 0 |
| PHC | 0.61 | 0 | 0 | 0.16 | 0 | 0.07 |
| TrT | 0 | -0.69 | 0.44 | -0.08 | 0 | -0.80 |
| **Occipital** |  |  |  |  |  |  |
| Cu | 0 | -0.06 | 0 | 0 | 0.06 | 0 |
| LO | 0 | 0 | 0 | 0 | 0 | 0.11 |
| Lg | 0.29 | 0 | 0 | 0.11 | 0 | 0.12 |
| PerCa | 0.28 | 0 | 0 | 0 | 0 | 0 |
| **Parietal** |  |  |  |  |  |  |
| IP | 0 | 0 | 0.1 | 0.03 | 0 | 0.17 |
| IstCg | 0.15 | -0.32 | 0 | 0 | 0 | 0 |
| PCC | 0 | -0.53 | 0 | 0 | 0.43 | 0 |
| PreCu | 0 | 0 | 0 | 0.18 | 0.67 | 0.19 |
| **Frontal** |  |  |  |  |  |  |
| PaC | 0 | -0.18 | 0.05 | 0.04 | 0.49 | 0.08 |
| Op | 0 | -0.12 | 0 | 0 | 0.01 | 0 |
| **Other** |  |  |  |  |  |  |
| Ins | 0 | -0.29 | 0.67 | 0 | 0 | -0.02 |
